# The moderating role of food cue sensitivity in the behavioral response of children to their neighborhood food environment: a cross-sectional study

**DOI:** 10.1186/s12966-017-0540-9

**Published:** 2017-07-05

**Authors:** Catherine Paquet, Luc de Montigny, Alice Labban, David Buckeridge, Yu Ma, Narendra Arora, Laurette Dubé

**Affiliations:** 1 Centre for Population Health Research, Sansom Institute for Health Research, School of Health Sciences, University of South Australia, GPO Box 2471, Adelaide, South Australia 5001 Australia; 2 Research Centre of the Douglas Mental Health University Institute, 6875 LaSalle Boulevard, Montreal, Québec H4H 1R3 Canada; 3 de Montigny Consulting, 3840 de l’Hôtel-de-Ville Ave., Montreal, Québec H2W 2G5 Canada; 4 Business Administration Division, Seaver College, Pepperdine University, 24255 Pacific Coast Hwy, Malibu, CA 90263-4237 USA; 5 Department of Epidemiology, Biostatistics and Occupational Health, McGill University, 1020 Pine Ave. West, Montreal, Québec H3A 1A2 Canada; 6 McGill Centre for the Convergence of Health and Economics (MCCHE), Faculty of Management, McGill University, 1001 rue Sherbrooke Ouest, Montreal, Québec H3A 1G5 Canada; 7 The INCLEN Trust International, F-1/5, 2nd Floor, Okhla Industrial Area Phase – 1, New Delhi, India

**Keywords:** Food environment, Food marketing, External eating, Children, Food consumption

## Abstract

**Background:**

Neighborhood food cues have been inconsistently related to residents’ health, possibly due to variations in residents’ sensitivity to such cues. This study sought to investigate the degree to which children’s predisposition to eat upon exposure to food environment and food cues (external eating), could explain differences in strength of associations between their food consumption and the type of food outlets and marketing strategies present in their neighborhood.

**Methods:**

Data were obtained from 616 6–12 y.o. children recruited into a population-based cross-sectional study in which food consumption was measured through a 24-h food recall and responsiveness to food cues measured using the external eating scale. The proportion of food retailers within 3 km of residence considered as “healthful” was calculated using a Geographical Information System. Neighborhood exposure to food marketing strategies (displays, discount frequency, variety, and price) for vegetables and soft drinks were derived from a geocoded digital marketing database. Adjusted mixed models with spatial covariance tested interaction effects of food environment indicators and external eating on food consumption.

**Results:**

In children with higher external eating scores, healthful food consumption was more positively related to vegetable displays, and more negatively to the display and variety of soft drinks. No interactions were observed for unhealthful food consumption and no main effects of food environment indicators were found on food consumption.

**Conclusions:**

Children differ in their responsiveness to marketing-related visual food cues on the basis of their external eating phenotype. Strategies aiming to increase the promotion of healthful relative to unhealthful food products in stores may be particularly beneficial for children identified as being more responsive to food cues.

**Electronic supplementary material:**

The online version of this article (doi:10.1186/s12966-017-0540-9) contains supplementary material, which is available to authorized users.

## Background

The ubiquitous presence of calorie-dense food and food cues in urban environments, be it in the absolute or in relation to healthier options, has been repeatedly blamed for the rising trends in overconsumption and associated obesity over the last few decades [1, 2]. For instances, within- and cross-country analyses have linked increases in the share of the population diet taken up by modern processed food typically rich in calories, fats and sugars with increases in obesity prevalence [3–5]. Beyond food per se, observational studies have linked changes in marketing practices, such as food advertising [6–8] and prices [9], to an obesogenic shift in consumption patterns. Other practices such as product packaging and portion sizes have similarly been associated with unhealthful diets [10–12].

Further evidence for the relationship between caloric excess/obesity and environmental exposure conditions comes from studies assessing the absolute or relative availability of food access points typically considered as *unhealthful* (e.g., fast food restaurants, convenience store) or *healthful* (e.g., supermarket, fresh food outlets) [13]. These studies tend to use geo-referenced store locations to determine the availability of food stores, most commonly within one’s neighborhood, be it defined using administrative units like census tracts, or unique geographical areas around the individual’s residence (or other reference points) using a pre-defined distance (e.g. 1-km or 1-mile radius). These studies have provided some evidence for associations between availability of fast-food stores or supermarkets and dietary and weight outcomes; however, null and unexpected findings are also common, especially in children (for reviews, see [14–18]). Most of these studies rely on the assumption that the presence of certain types of food outlets (e.g. supermarkets or fast food restaurants) is a reasonable proxy for availability of predominantly healthful or unhealthful food. It is however important to acknowledge that variation may exist in the type of food available within similar types of food outlets. For instance, some supermarkets may carry as much, if not more unhealthful food products, compared to healthful alternatives. A few studies have captured attributes of food products sold within outlets that are expected to influence purchase behaviors, such as shelf space, displays, price or promotions, and their associations with dietary and weight outcomes [19–21], which may provide a more accurate and comprehensive picture of neighborhood food cues than access-only models. These studies have mostly relied on store audits, with few capitalizing on the rich information available from digital marketing databases, which can provide a thorough, dynamic and comprehensive assessment of local food environment cues [22]. Only a minority of these studies have been conducted in children, with some evidence that children’s food consumption is related to fast-food and fruit and vegetable prices [23, 24]. Food marketing research has been mostly focused on television advertising with calls for more research on the impact of non-television food marketing [25], including sales promotions of energy-dense nutrition poor foods [26].

It is possible that the inconsistent findings on the relationship between neighborhood food environment and obesity at the population level are tied to the lack of consideration to putative individual differences that exist in one’s responsiveness to external exposure to food and food cues. Some evidence for individual differences in food responsiveness can be found in the neuroscience literature, especially from studies related to the dopamine pathways, which are thought to be key in guiding eating and other motivated behaviors in response to prevailing internal states and external cues, such as food pictures [27] and food advertisement [28, 29]. For instance, when comparing obese to lean adults or children, research demonstrated heightened response to food cues in “hedonic” regions receiving strong dopaminergic projections, such as the striatum, insula, amygdala, hippocampus and the orbitofrontal cortex, as well as altered response (often heightened) in prefrontal and cingulate cortices [30, 31], and altered feeding-related modulation of hedonic and executive region responses [32]. Lean youth at risk for future obesity by virtue of parental obesity have shown hyper-responsivity of reward regions to palatable food receipts [33]. Weight gains have been associated with hyper-responsivity to food intake [34], food images [35], and food commercials [36]. Laboratory studies examining eating behavior in children in response to controlled food environments (snack test) have also found similar results using either genetic markers or neurocognitive tasks related to differential dopamine signaling [37, 38]. Finally, a population-based observational study reported that a positive association between fast food consumption and neighborhood exposure to fast-food restaurants was only observed in adult participants with a high behavioral approach response [39], as measured by the behavioral approach system scale (BAS; [40]), a psychological scale reflecting one’s predisposition to incentives and appetitive stimuli, which has been linked to neural responses to food cues [41].

In this paper, we attempt to better characterize, in a population-based sample, children who may be more responsive to both unhealthful and healthful environmental food cues. A number of self-reported food-related trait measures have been developed over the years (for a review see [42]), many of which reflect the concept of uncontrolled eating [43]. In the present study, we examine external eating [44] as a food-specific index for individual difference in neurobehavioral environment responsiveness. External eating is a psychological measure that reflects one’s predisposition to eat upon exposure to environment food and food cues. This food-specific index of environment responsiveness has been associated with greater attentional bias and more positive evaluation of food cues [45], food cravings [46], and greater food consumption in response to food (vs neutral) commercials [47].

The aim of the study was to determine the degree to which external eating modifies the associations between measures of the food environment and food behavior in children, examining its effect on the consumption of both unhealthful food (i.e., nutrient poor, high fat/sugar/cal food) and healthful alternatives. It is hypothesized that healthful and unhealthful environmental cues related to the type of food outlets available within neighborhoods and to marketing strategies used within local stores (promotional activities and variety) would be more strongly associated with consumption in children with a higher sensitivity to external food cues.

## Methods

### Overview

Data for this cross-sectional study were obtained from a larger study (the “Brain-to-Society” study) which is examining multi-level (neighborhood, household, individual) drivers of childhood obesity. Parallel data collections targeting families with children age 6–12 were undertaken in Canada and India. This study focuses on the Canadian sample, which consisted of a convenience sample of households from the Montreal Metropolitan area. Recruitment and data collection was undertaken by an independent research marketing firm on behalf of the research team. Participants were randomly selected from a pre-established database of households identified as likely to have children in the target age group, and who had indicated their willingness to participate in academic research. This frame was provided by the research marketing firm.

A total of 4947 potential households were initially contacted by telephone and asked about their willingness to complete a survey about children’s eating and lifestyle habits. Of those, 813 were non-residential records and 633 were not eligible. A further 2352 had unknown eligibility. Of those eligible, 69 only partially completed the interview, 355 refused, 99 could not be recontacted, 10 were excluded due to gender quota and 616 completed the interviews for an estimated response rate of 23%, which accounts for a proportion of the unknown eligibility group that may be eligible. A﻿dditional information on sample and recruitment can be found in ﻿Electronic Additional file 1.

After confirming whether any child aged between 6 and 12 years old lived in the household, interviewers asked to speak with a parent/guardian with the best knowledge of the child’s daily habits, who was then asked to answer a series of questions regarding their child. When more than one eligible child lived in the household, the child with the next birthday was selected. Interviews were conducted between March and August 2013 and had a duration of 50 min. Participants received a CAD$10 incentive for their participation. Verbal consent was obtained from all participants included in the study, and ethical approval was obtained from McGill University’s Institutional Research Board.

### Measures

*Food consumption* was measured using a selective 24-h food recall in which parents/guardians were asked to identify the number of times their child consumed selected food items the day prior to the interview, from the time the child woke up to bed time. A selective 24-h recall was chosen to provide a time-efficient way to obtain an initial assessment of dietary intake during the phone interview, during which a range of measures was administered. A more complete 24-h recall was used in a follow up data collection on a reduced sample. The food items consisted of 15 food items and 12 drink options. Items were selected for their representativeness of children’s healthful and unhealthful dietary intake [48–50] in addition to cover food categories commonly targeted by health promotion and marketing activities and are provided in Electronic Additional file 2.

Two intake variables were created to capture the child’s consumption of healthful foods and unhealthful foods based on items expected to be sold in food stores. Healthful food consumption was based on the sum of the reported frequency of consumption of (1) whole grain foods, (2) fruits, (3) green vegetables, (4) other vegetables, and (5) water. Unhealthful food consumption was based on the sum of the reported frequency of consumption of (1) soft drinks, (2) salty snacks (e.g. chips, nachos, buttered popcorn), (3) candy package or chocolate bar, (4) cake, pie, cookies, doughnut, brownie, or other baked sweets, and (5) ice cream, ice cream bar, frozen yogurt, or popsicle. The two intake variables were compared to nutrient intake values computed from the more extensive 24-h dietary recall collected from a subset of the sample (*n* = 276). The healthful food consumption variable was correlated with fiber (*r* = 0.15, *p* = 0.01) and water (*r* = 0.12, *p* = 0.04) intake and the unhealthful food consumption variable was correlated with sucrose (*r* = 0.17, *p* = 0.006), carbohydrate (*r* = 0.12, *p* = 0.04), and fatty acid (*r* = 0.15, *p* = 0.01).

*External eating* was assessed using items from the Dutch Eating Behavior Questionnaire [44] as reported by parents. The ten items were re-worded for parents to report on their child’s behavior and adapted to fit the cultural context and improve suitability for children. For instance, the term delicious was replaced for yummy and food sources like cafes replaced for corner stores (‘dépanneur’). The number of response options were also changed from five to three consistent with previous adaptations of the scale for children [51]. Internal consistency of this scale for the sample, assessed following the approach recommended by Gadermann and colleagues [52] for estimating reliability coefficients for ordinal data, was found to be within the acceptable range, although not excellent (Cronbach’s alpha = 0.77).

#### Food environment

In order to provide a more comprehensive assessment of neighborhood food cues and consistent with calls for multi-dimensional measures of the neighborhood food environment that not only include access but also in-store food measures [53], two types of food environment measures were used in this study: the type of food retailers locally available and the marketing strategies used within local outlets. The types of food outlets locally available were measured using the *modified Retail Food Environment Index (mRFEI*), an index representing the proportion of food retailers classified as healthful developed by the Centers for Disease Control and Prevention (CDC) and used to standardize reports on community food environment [54]. The choice of a retail food environment index over availability of specific types of food stores is supported by evidence that combined indices are more robustly related to weight-related outcomes than individual food store availability measures [15]. Food outlets in the Montreal Census Metropolitan Area were identified using the Expanded Points of Interest (DMTI) database (2013). Field validation of this database has shown acceptable “representativity” of food outlets in the same city as the present study [55]. As per the CDC definition, supermarkets, grocery stores, fruit and vegetable stores, and supercenters were classified as “healthful”, and convenience stores and fast-food restaurants as “less healthful”. More information on the identification of food retailers can be found in Electronic Additional file 3. Food outlets and their locations were integrated into a Geographic Information System to identify all food outlets located within 3 km of each participant’s residence. The number of outlets was expressed using kernel density estimates (KDE; a distance-decay function weighing businesses closer to participants’ residence more heavily than those farther away) based on a 3-km circular buffer. A 3-km radius was selected based on results from a pilot analysis showing that over 99% of cohort home locations had one or more food outlets within this distance. The mRFEI was then calculated by dividing KDE value for “healthful” food outlets by the sum of the KDE values for “healthful” and “less healthful” food outlets. This proportion was then multiplied by 100 to obtain a percentage of outlet density categorized as healthful.

#### Food marketing strategies

It is well known from the consumer behavior literature (for a review [56]) that business practices grouped under the umbrella of the four “P’s” of marketing (Product/Place/Price/Promotion) impact behavior in general, including food consumption. While the above mRFEI measure taps into the type and location of products at the community level, it fails to address price and promotion strategies as well as the range of products available. We therefore investigated two types of promotional activity (frequency of price-promotion and food displays), regular price, and variety of food products available within selected healthful and unhealthful food categories. Promotional activities and variety were expected to act as visual food-related environmental cues. Regular price was included as a strategy that would not necessarily be expected to act as visual food cue and was included as control strategy.

These measures were derived from a digital marketing database and have been described elsewhere [22]. In short, data for every single Universal Product Code (UPC) within target categories sold within stores located in the province of Québec, Canada were obtained from data on weekly purchases and marketing activities related to consumer packaged goods in stores (ScanTrack) purchased from the Nielsen Corporation for the period of 2008–2013. Retailers consisted of grocery stores, mass merchandisers and convenience stores. Their locations were represented using forward sortation areas (FSA), which are geographic regions defined by the first three digits of Canadian postal codes, and linked to participants based on their residential postal code. For each UPC, the following information was available: weekly price and in-store promotion, item description, brand name, package size, and the number of individual items within the pack. Food and Drug Administration (FDA) data on average serving size and unit package size were used to derive number of servings per unit.

Regular price, frequency of price promotions, variety and food display indicators were selected for the following product categories: regular soft drinks and vegetables. Both categories were selected due to their established links with weight, obesity and cardio-metabolic diseases [57–61] and the considerable focus of public health policies and interventions on these food categories. *Regular price* per serving was derived from the weighted average of the highest price of each UPC within the category over a three-month moving window [62]. *Frequency of price promotion* (discounts) category was obtained from the weighted average number of weeks in which the price of a given UPC was at least two standard deviations below its average price [63]. In both cases, overall market shares of each UPC in the entire period (2008–2013) within their category were used as weights. *Non-price promotion (food displays)* was operationalized as the proportion of food products (UPCs) within a target food category that was on display (e.g. end of aisles displays) in a given week in a given store. *Variety* was operationalized as the average number of different Stock Keeping Unit (SKU; a number that uniquely identifies distinct products (and package sizes)) within product categories weighted by stores monthly sales. Marketing indicators data collected during the 12 months preceding participants’ recruitment were averaged and used as a measure of marketing exposures in analyses.

### Analyses

Generalized linear mixed models were used to model, separately, children’s healthful or unhealthful food consumption as a function of food environment measures, external eating and their interactions. Models were estimated with exponential spatial covariance structure, where the covariance between two observations depended on the distance between the two observations. Linear models were used for healthful food consumption, whose distribution was approximately normal and Poisson models were used for unhealthful food, whose distribution was positively skewed. Food environment measures were modelled separately to maximize sample size for each food environment measure and to avoid multiple interaction terms in the same model. External eating and environmental variables were standardized prior to analyses. All analyses accounted for socio-demographic characteristics such as children’s age, gender, and household income (0-45 K,45-65 K, and >65 K CAD), and language in which the interview was done (French/English, proxy for cultural background). Area-level median household income and employment rates were initially considered as covariates but not included due to lack of associations with outcome variables. Statistical significance was set at alpha = 0.05, but interactions terms with level of significance up to alpha = 0.10 are also discussed considering the recognized lower statistical power of interaction testing [64], especially in non-experimental research [65].

Different sample sizes were available for the different exposure variables, with marketing indicators being available for fewer participants compared to the relative availability of food outlet indicator. In order to capitalize on all information available, each analysis was conducted using the maximum sample size available. In order to minimize the chance of bias due to missing marketing data, the odds of participants having a missing marketing indicator was modelled in relation to behavioral outcomes and all environment-unrelated predictors, population density (2011 Census Data) and socio-economic characteristics of the Census Tract of residence (% high school completion, median household income, prevalence of low income, % immigrants, employment rate (2006 Census Data (not available from 2011 Census)). Statistically significant predictors of missing marketing data (employment rate, median household income, and participant’s gender for soft drink indicators, and % high school completion and population density for vegetable marketing indicators) were used to obtain probability of missing marketing data and the inverse of this probability was used as weight in their respective analyses (inverse probability weighting).

## Results

### Descriptive statistics

Of the 616 children recruited in the study, two had missing food consumption information and 30 had missing household income information. Eight participants had a missing mRFEI, which resulted in a sample size of 576 children. Marketing data on regular soft drinks were available for 465 participants whereas vegetable data were available for 257 participants. The lower numbers of observations for vegetables were due to absence of sales from this food category in stores sampled one year prior to recruitment. Socio-demographic profiles of the analytic samples for the mRFEI, soft drink marketing, and vegetable marketing indicators are provided in Table 1 along with descriptive statistics on outcome and exposure variables. Comparisons of soft-drink marketing variables across the two marketing indicator analytic samples suggested that the vegetable marketing sample had lower soft drink prices, greater number of displays and variety, and more frequent discounts suggesting a possible greater representation of larger stores (e.g. supermarkets) compared to smaller stores. No differences in socio-demographic or behavioral profile were found across the three analytical samples.

**Table 1:** Descriptive statistics for analytic samples

	mRFEI analysis	Marketing strategy analyses	
	(*n* = 576)	Soft drinks (*n* = 465)	Vegetables (*n* = 257)
Age (mean(SD))	9.1 (1.7)	9.1 (1.7)	9.0 (1.6)
Gender (n(%) boys)	284 (49.3%)	217 (46.7%)	109 (42.2%)
Household Income (n(%))			
< 45 K	111 (19.3%)	96 (20.6%)	48 (18.6%)
45–65 K	114 (19.8%)	94 (20.2%)	45 (17.4%)
> 65 K	351 (60.9%)	275 (59.1%)	165 (64.0%)
Language survey conducted (n (%) French)	320 (55.6%)	256 (55.1%)	149 (57.7%)
Healthful eating score (mean (SD))	8.6 (3.6)	8.7 (3.6)	8.7 (3.6)
Unhealthful food score (mean (SD))	1.6 (1.2)	1.6 (1.2)	1.6 (1.3)
External eating score (Range: 10–30; mean (SD))	21 (4)	21 (4)	21 (4)
mRFEI (mean (SD))	23.3 (11.7)	22.4 (10.5)	22.4 (10.7)
Soft drinks discount frequency (mean (SD))		0.41 (0.19)^a^	0.50 (0.15) ^a^
Soft drinks display (mean (SD))		0.26 (0.22) ^a^	0.38 (0.17) ^a^
Soft drinks regular price per serving (mean (SD))		0.37 (0.12) ^a^	0.30 (0.04) ^a^
Soft drinks variety (mean (SD))		128.2 (60.3) ^a^	171.8 (44.7) ^a^
Vegetable discount frequency (mean (SD))			0.47 (0.13)
Vegetable display (mean (SD))			0.05 (0.04)
Vegetable regular price per serving (mean (SD))			0.31 (0.08)
Vegetable variety (mean (SD))			410.5 (116.7)

*mRFEI* modified Retail Food Environment Index (proportion of food retailers classified as healthful), *SD* Standard Deviation

^a^ mean difference statistically significant (*p* < 0.05)

### Relative availability of healthful food outlets

Results related to the availability of healthful food outlets are reported in Table 2. No main or interactive effects of the food environment was observed, but a statistically significant positive association was found between external eating and unhealthful food consumption.

**Table 2:** Main and interactive effect of external eating and mRFEI on food consumption

Predictors	Healthful food consumption			Unhealthful food consumption		
	Estimate	95% CI	*P*	RR	95% CI	*P*
External eating score (1 SD)	−0.18	(−0.49, 0.12)	0.23	1.09	(1.03, 1.16)	0.004
mRFEI (1 SD)	−0.05	(−0.35, 0.25)	0.75	0.98	(0.92, 1.04)	0.49
mRFEI x external eating score	0.24	(−0.06, 0.54)	0.11	1.00	(0.94, 1.06)	0.87

Results of regression models of healthful and unhealthful food consumption predicted by external eating status, mRFEI and their interactions, adjusted for child age, gender, language household income, and population density (*n* = 576), *RR* relative risk, *CI* Confidence Interval, *mRFEI* modified Retail Food Environment Index (proportion of food retailers classified as healthful), *SD* Standard Deviation

### Marketing food strategies

Results of analyses on the discount frequency, display, regular price, and variety of regular soft drinks are presented in Table 3. Similar to results related to the relative availability of healthful food outlets, no main effects of food environment indicators were found, but external eating was found to be positively related to unhealthful food consumption. No interaction effects between food environment indicators and external eating reached the 0.05 statistical significance level. Marginally significant interactions were, however, observed for soft drink display and variety in relation to healthful food consumption, with both interaction terms being negative suggesting that associations between these soft drink marketing indicators and healthful food consumption may be more negative for participants with higher external eating scores.

**Table 3:** Main and interactive effect of external eating and marketing indicators for soft drinks on food consumption

	Healthful food consumption			Unhealthful food consumption		
	Estimate	95%CI	*P*	RR	95%CI	*P*
External eating (1 SD)	−0.25	(−0.58, 0.07)	0.14	1.10	(1.03, 1.18)	0.005
Soft drink Discount Frequency (1 SD)	0.13	(−0.21, 0.47)	0.45	1.01	(0.94, 1.08)	0.87
External eating x Discount frequency	−0.22	(−0.54, 0.11)	0.20	0.98	(0.92, 1.05)	0.57
External eating (1 SD)	−0.26	(−0.59, 0.06)	0.11	1.10	(1.03, 1.18)	0.007
Soft drink Display (1 SD)	0.12	(−0.23, 0.48)	0.49	1.00	(0.92, 1.07)	0.91
External Eating x Display	−0.33	(−0.67, 0.01)	0.057	0.97	(0.90, 1.04)	0.39
External eating (1 SD)	−0.24	(−0.57, 0.09)	0.15	1.10	(1.03, 1.18)	0.005
Soft drink Regular price (1 SD)	0.04	(−0.32, 0.39)	0.84	0.97	(0.90, 1.05)	0.49
External Eating x Regular Price	0.07	(−0.24, 0.40)	0.65	1.01	(0.94, 1.08)	0.85
External eating (1 SD)	−0.25	(−0.58, 0.07)	0.09	1.10	(1.03, 1.18)	0.007
Variety (1 SD)	−0.01	(−0.36, 0.37)	0.97	0.98	(0.91, 1.06)	0.62
External eating x Variety	−0.31	(−0.65, 0.03)	0.076	0.95	(0.88, 1.02)	0.17

Results of regression models analysis testing the interactive effect of external eating and marketing indicators for soft drinks on healthful and unhealthful food consumption (*n* = 465) adjusted for child age, gender, language, and household income. *RR* relative risk, *CI* Confidence Interval, *SD* Standard Deviation

Results of analyses on the display, regular price, variety and discount frequency of vegetables are presented in Table 4. As for the analyses for the relative availability of healthful food outlets and soft drink marketing indicators, no main effects of marketing indicators were observed. Evidence of interactions between external eating and vegetable displays was found for healthful food consumption. The interaction term was positive, suggesting that a positive association between vegetable display and healthful food consumption might be more positive for children with higher external eating scores. The association between external eating and unhealthful food consumption observed previously did not reach statistical significance in this reduced sample.

**Table 4:** Main and interactive effects of external eating and marketing indicators for vegetables on food consumption

	Healthful food consumption			Unhealthful food consumption		
	Estimate	95%CI	*P*	RR	95%CI	*P*
External eating (1 SD)	−0.42	(−0.96, −0.01)	0.07	1.08	(0.97, 1.20)	0.17
Vegetables Discount Frequency (1 SD)	0.13	(−0.18, 0.72)	0.54	0.99	(0.90, 1.10)	0.89
External eating x Discount frequency	−0.30	(−1.15, −0.09)	0.26	0.95	(0.85, 1.07)	0.42
External eating (1 SD)	−0.39	(−0.84, 0.06)	0.09	1.08	(0.97, 1.20)	0.15
Vegetables Display (1 SD)	−0.11	(−0.57, 0.35)	0.64	0.95	(0.85, 1.06)	0.35
External Eating x Display	0.55	(0.06, 1.04)	0.027	1.02	(0.91, 1.15)	0.71
External eating (1 SD)	−0.42	(−0.88, 0.04)	0.07	1.08	(0.97, 1.20)	0.16
Vegetables Regular price (1 SD)	−0.01	(−0.44, 0.42)	0.95	1.00	(0.91, 1.11)	0.92
External Eating x Regular Price	−0.22	(−0.83, 0.40)	0.49	0.94	(0.82, 1.08)	0.39
External eating (1 SD)	−0.03	(−0.94, 0.89)	0.95	1.19	(0.97. 1.46)	0.09
Vegetable Variety (1 SD)	0.21	(−0.67, 1.09)	0.64	0.97	(0.80, 1.19)	0.79
External Eating x Variety	−0.47	(−1.41, 0.48)	0.33	0.88	(0.71, 1.09)	0.25

Results of regression models separately testing the main and interactive effects of external eating and marketing indicators for vegetables (*n* = 257) on health and unhealthful food consumption adjusted for child age, gender, language, and household income. *RR* Relative risk, *CI* Confidence Interval, *SD* Standard Deviation

### Visualization of interaction effects

**Fig. 1:**
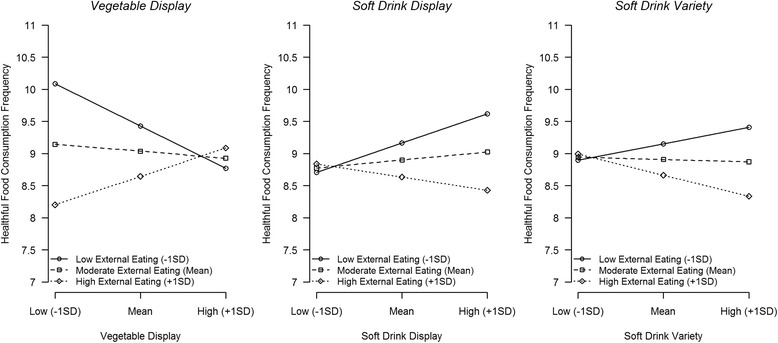
Predicted healthful food consumption frequency for high/moderate/low values of external eating and food environment indicators (predicted values for “average participant”, namely a girl 9.1 years of age, from a French-speaking household with household income of CAD$45-65 K)

Consumption frequency predicted from models in which statistically significant, or marginally significant interactions were found were plotted for different levels (mean ± one standard deviation) of the food environment indicators and external eating scores (see Figure 1). High external eaters seemed to consume less healthful food compared to low or intermediate external eaters in environments that can be considered less healthful, with a difference in daily healthful food consumption frequency of 1.9 in environments with low vegetable displays, and 1.2 and 1.1 in environments with high soft drink displays and variety, respectively. No differences in consumption were apparent for values of the food environment considered more healthful (higher vegetable display and lower soft drink variety and display).

## Discussion

This study investigated variations in the strength of association between various aspects of the neighborhood food environment and food consumption in children as a function of individual differences in responsiveness to food cues, as measured using the external eating scale. No evidence was found for a moderating influence of external eating on the relationship between the relative availability of healthful, compared to unhealthful food retailers in proximity to a child’s residence, and children’s diet. However, some evidence for a moderating effect of external eating was found when looking at marketing practices related to visual food cues such as store displays used within food outlets to promote healthful (vegetables) and unhealthful foods (soft drinks), and variety of soft drink products. Specifically, the direction of the effect of store displays and soft drink variety for children with greater external eating was consistent with the dietary quality of each category (more healthful eating for vegetables and less healthful food consumption for soft drinks). These results are consistent with a laboratory-based study conducted with adults showing that the impact of viewing a food commercial (compared to a neutral commercial) while watching television was only observed in high, but not low, external eaters [47].

Our results, if replicated, would suggest that display strategies may be important environmental cues for children with external eating tendencies. Our findings would suggest that external eaters in this sample may not only respond to high-fat and high-sugar foods, but also to other types of food when they are more present in their immediate environment. The fact that external eating is associated with better diets in children when exposed to healthful food environment is consistent with the proposition that external eating might be adaptive in children [66, 67] and potentially explain negative or null correlation found between external eating and body mass index [66, 68].

No evidence of main or interactive effects of regular price or discount frequency was found. Regular pricing was not expected to lead to selected attention to particular food products, especially in the targeted age group and was included as a control strategy. Although discount frequency might be associated with greater product visibility, it remains mainly a financial purchasing incentive, which may not be a strong incentive for children. The null finding for pricing, and to some degree discount frequency, therefore supports the specificity of the results for strategies that are associated with specific environmental visual cues.

A main effect of external eating, but no interactions with food environment measures, were found for unhealthful food consumption. It is possible that unhealthful foods, with their high rewarding values and their omnipresence in urban environments, may have reached a “visibility threshold” so that additional variance in their availability is insufficient to translate into behavioral differences, even for high external eaters. The relative overrepresentation of unhealthful foods is supported with our data where only 23% of food outlets were considered as a source of healthful food (as suggested by the mRFEI) and where in-store displays of soft drinks were also much more frequent than displays of vegetables.

The lack of main effect of marketing indicators on either food consumption measures contrasts with earlier work analyzing store sales data [69] showing that the same reinforcing marketing practices that increase sales of unhealthful food (namely soft drinks), when applied to healthful food (namely vegetables), were also successful in increasing their sales. Discount frequency results also contrast with findings from a recent study showing that the increase in sales associated with frequency of price discounts was largest for less healthy compared to healthier food categories [70]. However, when testing whether responsiveness to price discounts of the healthy and less healthy food categories varied by SES status, evidence for a SES gradient was only found for healthy compared to less healthy categories, a similar pattern to what was found in this study. It must be noted that the above results are based on sales data, which generally provide large sample sizes, but limited information on individuals beyond basic demographics as well as an incomplete representation of individual diets.

Our findings suggest high responsiveness to food cues, while tied to potential adverse consequences when exposed to unhealthful environments, may translate into a beneficial impact in more healthful environments. This interpretation is also consistent with recent evidence that children carrying the 7-repeat allele on DRD4 (reflecting genetic predisposition to high environmental responsiveness) from a disadvantaged socio-economic background consumed more fat than the average, while the same carriers who were, in contrast, coming from a more advantaged socio-economic background consumed less fat than the average [71, 72]. That study concluded that the genetic marker identified individuals with “differential susceptibility” to socio-economic disadvantage, which has also been linked to the nutritional quality of the food environment.

### Limitations

In this study, it was assumed that neighborhood food environment would influence children dietary behavior through children’s influence on parents’ purchases. It could be argued that children’s responsiveness to these food cues is unlikely to influence food behavior in this age group, who may not play an active role in household food purchasing decisions. However, evidence exists that children play an important role in supermarket product purchases [73, 74], and that younger children may be more likely to ask for advertised food products compared to adolescents [75]. Food marketing is seen as a predictor of food preference and attitude in children [25, 76], even in children as young as 5–6 years old [77]. This was reflected in this sample, for which a subsample (*n* = 379) of parents were asked questions specifically related to the role of children in purchasing decisions, with 85% stating that they would never, or rarely, *not* allow their children to ask for products and only 17% that would never or rarely tell their children they would consider their preferences when making purchases.

It is also possible that children’s external eating tendencies are confounded by their parents’ own external eating behaviors or other type of parental influence. This is supported by evidence that maternal external eating is related to external eating of daughters and sons [78] and external eating in children is positively associated with perceived parental pressure to eat in boys and negatively associated with perceived parental restriction to eat in boys and girls [79]. Future research should aim to account for parental factors in these relationships. In addition, parents may not have accurately reported their child’s intake or external eating tendencies, especially for older children, and their reports may have been influenced by a social desirability bias. Moreover, the food consumption instrument focused only on a selection of food items and did not provide a comprehensive assessment of the child’s diet. The cross-sectional nature of the study precludes any causal inference and future studies should explore how these associations evolve over time. The relatively small sample size available for some analyses, especially those involving strategies to promote vegetable purchases, as well as the relatively low reliability of the external eating scale, resulted in limited statistical power for detecting interaction effects. In addition, food marketing variables were only available at a relatively large area level, which may not be a very representative picture of children’s most immediate food environment. However, a recent review comparing food environment associations across different expressions of neighborhood suggested that more immediate food environment may not be as important as food environment defined for larger surroundings [15]. In order to assess the separate influence of healthful and unhealthful food marketing and compare ‘visual’ strategies like displays with more economic strategies like pricing and discounts, a relatively high number of interactions were tested. This, combined with the use of a statistical significance level of 0.10, may have increased the risk of chance findings. However, consistency in the type of behaviors and strategies for which statistical significance was found, as well as the absence of associations for the ‘control’ strategy do not suggest that results are due to chance alone. Finally, the convenience sample used limits the generalizability of our results. Future studies should replicate findings in other populations.

## Conclusion

Findings from this study suggest that marketing strategies used to promote healthful and unhealthful food products within local stores may be more strongly associated with food consumption in children who were identified as being more responsive to food cues. These children’s intake appeared to be related to healthful and to some degree unhealthful food cues. These results, if replicated, would suggest that the development of interventions that simultaneously aim to reduce unhealthful local food cues while increasing healthful food cues may have a double effect in this subpopulation. Findings indicated that, in this sample, not all types of food cues interacted with external eating tendencies, with “visual cues” like in-store displays being more consistently related to healthful food consumption than “economic” cues like price or discounts in high-external eating children. If replicated, the results of this study may inform the development of targeted strategies promoting healthier food choices that are likely to have a greatest impact in specific sub-populations. Future studies should explore other measures of sensitivity to food cues measured through a variety of markers (genetic, neurocognitive, psychological) and their potential to identify responsiveness to a similar range of environmental healthful and unhealthful food cues, as well as clinical outcomes like weight status.

## Additional files

Additional file 1: Additional information on sample and recruitment. (DOCX 16 kb)

Additional file 2: Food items included in 24-hour recall. (DOCX 15 kb)

Additional file 3: Food outlet classification. (DOCX 16 kb)

## Abbreviations

CAD: Canadian dollars
FDA: Food and Drug Administration
FSA: Forward sortation areas
mRFEI: modified retail food environment index
SIC: Standard Industry classification
SKU: Stock keeping unit
UPC: Universal product code
