# Supplementary material for: The moderating role of food cue sensitivity in the behavioral response of children to their neighborhood food environment: a cross-sectional study
Source: Int J Behav Nutr Phys Act. 2017 Jul 5;14:86. doi: 10.1186/s12966-017-0540-9 (PMC5499022; doi:10.1186/s12966-017-0540-9)
Supplement: Supplementary file 1 — Additional information on sample and recruitment. (DOCX 16 kb) [file 12966_2017_540_MOESM1_ESM.docx]

**Additional File 1**

**Additional information on sample and recruitment**

Participants were randomly selected from a list created based on the conduct of previous surveys by the research firm where households with children age 6 to 12 who lived in the Montreal Census Metropolitan Area and who also agreed to participate in future research were identified and provided a telephone number and mailing address. In addition, to be eligible, households had to have the child residing in their household most of the time. For households with more than one child in the targeted age range, interviewers asked parents to answer the questions keeping in mind the child whose birthday next followed in the calendar year.

Unfortunately, we do not have information on households from the general population that would meet the inclusion criteria used to create the above sampling frame, nor do we have sufficient information from participants in the sampling frame to do a direct comparison to assess representativeness of the sample. Loss of sample from the recruited sample (n=616) to the largest analytical sample (n=576, mRFEI sample) was due to missing information on household income (n=30), food consumption (n=2), and mRFEI (n=8). This analytical sample did not differ from the recruited sample (see table below). Comparisons between the different analytical samples are provided in Table 1 in the manuscript.

|  | Recruited sample | mRFEI analysis |
| --- | --- | --- |
|  | N=616* | (n=576) |
| Age (mean(SD)) | 9.1 (1.7) | 9.1 (1.7) |
| Gender (n(%) boys) | 307 (50.1%) | 284 (49.3%) |
| Language survey conducted (n (%) French) | 341 (55.3%) | 320 (55.6%) |
| Healthful eating score (Mean (SD)) (n=614) | 8.6(3.6) | 8.6 (3.6) |
| Unhealthful food score (Mean (SD)) | 1.6 (1.2) | 1.6 (1.2) |
| External eating score (Range: 10-30; Mean (SD)) | 21 (4) | 21 (4) |
| mRFEI (mean (SD)) | 23.1 (11.7) | 23.3 (11.7) |

*n=614 for food scores, n=608 for mRFEI

Participants with missing information on predictors and covariates were excluded from the analytical sample. As discussed in the paper, missing information was more significant for analyses using marketing indicators and inverse probability weighting was used for these specific analyses.
