# Supplementary material for: The moderating role of food cue sensitivity in the behavioral response of children to their neighborhood food environment: a cross-sectional study
Source: Int J Behav Nutr Phys Act. 2017 Jul 5;14:86. doi: 10.1186/s12966-017-0540-9 (PMC5499022; doi:10.1186/s12966-017-0540-9)
Supplement: Supplementary file 2 — Food items included in 24-hour recall. (DOCX 15 kb) [file 12966_2017_540_MOESM2_ESM.docx]

**Electronic Additional File 2**

**Food items included in 24-hour recall**

YESTERDAY, from the time your child woke up until the time he or she went to bed, **how many servings** of the following did your child drink?

INTERVIEWER NOTE: *a drink serving is one cup, one small bottle, one can, or one drinking box (e.g. can of pop, a small bottle of water, a medium coffee, a small carton of milk, or a drinking box of juice).*

|  | Number of servings | | | | | | |
| --- | --- | --- | --- | --- | --- | --- | --- |
|  | 0 | 1 | 2 | 3 | 4 | 5 | 6+ |
| White or chocolate milk or soy beverages |  |  |  |  |  |  |  |
| 100% fruit juice or vegetable juice |  |  |  |  |  |  |  |
| Fruit-flavored drinks (Kool-Aid, Sunny D or lemonade…etc) |  |  |  |  |  |  |  |
| Soft drinks or diet pop |  |  |  |  |  |  |  |
| Sports drinks (Gatorade) |  |  |  |  |  |  |  |
| High energy drinks (Red Bull) |  |  |  |  |  |  |  |
| Hot Chocolate, cappuccino, Frappuccino |  |  |  |  |  |  |  |
| Tea, iced tea, or coffee |  |  |  |  |  |  |  |
| Slurpees, slushies, or snow cones |  |  |  |  |  |  |  |
| Shakes |  |  |  |  |  |  |  |
| Water |  |  |  |  |  |  |  |
| Flavored Water (Vitamin Water, Crystal Light) |  |  |  |  |  |  |  |

YESTERDAY, from the time your child woke up until the time he or she went to bed, **how many times** did your child eat the following foods?

|  | Number of times | | | | | | |
| --- | --- | --- | --- | --- | --- | --- | --- |
|  | 0 | 1 | 2 | 3 | 4 | 5 | 6+ |
| Salty snacks (e.g. chips, nachos, buttered popcorn) |  |  |  |  |  |  |  |
| Lentils, chickpeas (hummus), kidney beans, or other dried beans |  |  |  |  |  |  |  |
| Fish or shellfish |  |  |  |  |  |  |  |
| One slice of pizza or a pizza snack |  |  |  |  |  |  |  |
| One hot dog or sausage on a bun |  |  |  |  |  |  |  |
| One hamburger or cheeseburger |  |  |  |  |  |  |  |
| One sub or deli sandwich |  |  |  |  |  |  |  |
| Whole grains |  |  |  |  |  |  |  |
| Fruit, not including juice |  |  |  |  |  |  |  |
| Dark green vegetables |  |  |  |  |  |  |  |
| Other vegetables |  |  |  |  |  |  |  |
| French fries or other fried potatoes |  |  |  |  |  |  |  |
| One package of candy or one chocolate bar |  |  |  |  |  |  |  |
| One slice of cake or pie, two cookies, one doughnut, one brownie, or other baked sweets |  |  |  |  |  |  |  |
| Ice cream, an ice cream bar, frozen yoghurt, or a Popsicle |  |  |  |  |  |  |  |
