# Supplementary material for: The moderating role of food cue sensitivity in the behavioral response of children to their neighborhood food environment: a cross-sectional study
Source: Int J Behav Nutr Phys Act. 2017 Jul 5;14:86. doi: 10.1186/s12966-017-0540-9 (PMC5499022; doi:10.1186/s12966-017-0540-9)
Supplement: Supplementary file 3 — Food outlet classification. (DOCX 16 kb) [file 12966_2017_540_MOESM3_ESM.docx]

**Additional File 3**

**Food outlet classification**

The *modified Retail Food Environment Index (mRFEI)* is a ratio measure of healthful food outlets over the sum of healthful and less healthful food outlets. Following the Centers for Disease Control and Prevention (CDC) classification, supermarkets, grocery stores, fruit and vegetable stores, and supercenters were classified as healthful and convenience stores, fast-food restaurants as less healthful. Food outlets were classified into the CDC categories using a combination of Standard Industry Classification (SIC) 4-digit codes and keyword matching on business name, with the exception of supercenters which were identified by name only. Codes, keywords and/or names used for each category are presented below.

1. **“Healthful” outlets**
   1. **Supermarkets; grocery stores.** The DMTI datasets did not allow us to differentiate between “small” and “large” grocery stores as per the CDC definition of healthful and less healthful food retailers. We considered all grocery stores to be healthful, while focusing on differentiating between convenience stores and grocery stores. The 5411 SIC code (Grocery Stores) was used in combination with the following keywords:
      - INCLUDE: A&P, Adonis, Ag Foods, Alimentation, Askew's, Atlantic Superstore, Atlantic Supervalu, Avril, Axep, Barn, Boni Choix, Bulkley Valley Wholesale, Buy Low Foods, Coleman's, Commisso's Food, Cooper's Foods, Co Op Atlantic, Co Op West, Darrigo's, Dipietro's, Dominion, Dutch Boy, Econo Mart, Extra Foods, Family Foods, Farm Boy, Food Basics, Food City, Food Land, Food Town, Foodex, Foodfare, Fortinos, Freshco, Freshmart, Galati Brothers, Gordons, Grocer, Epicerie, H Mart, Highland Farms, Hylouie, Inter March, Knechtel Foods, Knob Hill Farms, Lalumiere Bonanza, Jardin Mobile, Marche Vegetarien, 5 Saisons, Arpents Verts, Entrepots Presto, Loblaw, Loeb, Lofood, Longo's, Markets, Marches, Mike Dean's Super Food Stores, Miracle Food Mart, Nature's Emporium, No Frills, Northmart, Ok Economy, Overwaitea Foods, Pat Mart, Penner Foods Manitoba, Piggly Wiggly, Price Chopper, Pricesmart Foods, Provigo, Quality Foods, Rachelle Bery, Real Canadian Wholesale Club, Red Rooster, Richelieu, Safeway, Saveeasy, Save On Foods, Servi Express, Sobey, Supermarket, Supermarche, Supervalu, Super C, T&T, Taiko Supermarket, The Barn Fruit Markets, Real Canadian Superstore, Thrifty Foods, Tomboy, Ultra Food & Drug, Urban Fare, Valdi, Valu Foods, Valu Mart, Village Food Stores, White's, Whole Foods, Zehrs, Iga, Maxi, Metro
      - EXCLUDE: Depanneur
   2. **Produce stores.** The 5431 SIC code (Fruit and Vegetable Markets) was used in combination with the following keywords:
      - EXCLUDE: Cafe, Caterer, Traiteur, Confiserie, Chocolat, Livraison, Delivery, Fleur, Flower
   3. **Supercenters**: The following keywords were used to identify supercenters:
      - INCLUDE: Wal Mart, Costco, Target (The DMTI datasets did not include records for Target supercenters).
      - EXCLUDE: Pneu, Tire, Information, Opti, Photo, Service, Restaurant, Greiche, Pharma, Comptoir, Sleep, Centre, Center, Galer, Succursale
2. **“Less healthful” outlets**
   1. **Fast Food Restaurants:** The 5812 SIC code (Eating Places) was used in combination with the following keywords:
      - INCLUDE: 2-For-1, A&W, Amir, Arby, Ashton, Basha, Belle Province, Blimpie, Booster Juice, Buffalo Bill, Burger, Submarine, Carl's Jr, Casse Croute, Chez Ashton, Chez Gerard, Chicken Delight, Chick'n'chick, Cinnabon, Coffee Time, Country Style, Croissant Plus, Dagwoods, Dairy Queen, Dic Ann's, Dixie Lee Fried Chicken, Dixie Lee, Edo Japan, Extreme Pita, Fast Eddies, Fast Food, Frank Supreme, Frit, Frie, Fryer's, Goji's, Greco Pizza, Harvey, Ho Lee Chow, Hot Dog, Jimmy The Greek, Jugo Juice, Kfc, Pfk, Kojax, Krispy Kreme, La Belle Province, Lafleur, Los Dias, Mac Donald, Manchu Wok, Mary Brown's, Mike, Monsieur Falafel, Mr Sub, Orange Julius, Patate, Potato, Patio Vidal, Pik Nik, Pita Pit, Pizz, Place Tevere, Popeyes Louisiana Kitchen, Poutine, Pretzel, Quizno, Saint Hubert, Sous Marin, Stratos, Sub Way, Swiss Chalet, Taco Bell, Taco Time, Teriyaki Experience, Thai Express, Thai Zone, Tiki Ming, Tim Hortons, Valentine, Wendy, White Spot, Williams Fresh Cafe, Yogen Fruz.
      - EXCLUDE: Pneu, Tire, Information, Opti, Photo, Service, Restaurant, Greiche, Pharma, Comptoir, Sleep, Centre, Center, Galer, Succursale
   2. **Convenience Stores** The 5411 SIC code (Grocery Stores) was used in combination with the following keywords:
      - INCLUDE: Essence, Esso, Shell, Ultramar, 7 Eleven, 7 Jours, Beau Soir, Becker, Boni Soir, Bonus, Convenien, Couche Tard, Depanneur, Duritas, Husky, On The Run, Petro, Pioneer Energy, Provi Soir, Quik, Quick, Gas, Service Station, Shell, Short Stop, Station Service, Tabagie, Variety.
      - EXCLUDE: Dollar

.
